# An enzyme-responsive double-locked amonafide prodrug for the treatment of glioblastoma with minimal side effects†

**DOI:** 10.1039/d4sc04555f

**Published:** 2024-11-01

**Authors:** Wei Cheng, Yanli Yang, Bo Zhang, Chen-Wen Shao, Wei Chen, Ruimin Xia, Wenwei Sun, Xiubo Zhao, Bing Zhang, Xiangjie Luo, Tony D. James, Yong Qian

**Affiliations:** a Jiangsu Collaborative Innovation Center of Biomedical Functional Materials, School of Chemistry and Materials Science, Nanjing Normal University Nanjing 210046 China yongqian@njnu.edu.cn; b School of Pharmacy, Changzhou University Changzhou 213164 China zhangbo1027@cczu.edu.cn; c Department of Radiology, Nanjing Drum Tower Hospital, The Affiliated Drum Tower Hospital of Nanjing University Medical School Nanjing 210008 China; d Wuxi School of Medicine, Jiangnan University Wuxi 214122 China; e Department of Chemistry, University of Bath Bath BA2 7AY UK T.D.James@bath.ac.uk; f School of Chemistry and Chemical Engineering, Henan Normal University Xinxiang 453007 China

## Abstract

Amonafide (ANF), a topoisomerase II inhibitor and DNA intercalator, has exhibited promise in phase II trials but faces significant limitations due to adverse side effects. Here, we have developed a novel enzyme-triggered fluorogenic prodrug, AcKLP, that incorporates dual-locked enzyme activation, ensuring that the prodrug remains inactive until it confronts the unique enzymatic environment of glioblastoma cells. This approach minimizes premature activation and reduces toxicity to normal cells, with an IC_50_ > 100 μM for human umbilical vein endothelial cells (HUVEC) and ∼2.3 μM for human glioblastoma cells (U87). Upon activation of AcKLP by two distinct enzymes prevalent in glioblastoma cells, amonafide is released and emits a fluorescence signal response, facilitating treatment and the monitoring of real-time drug distribution. Mechanistic studies indicate that AcKLP mainly induces autophagic cell death in U87 cells. Moreover, three-dimensional multicellular U87 tumor spheroid assays and *in vivo* experiments confirm the potent antiproliferative activity of AcKLP against glioblastoma cells. This work demonstrates a novel de-caging strategy to improve the selectivity and efficacy of amonafide for cancer therapy.

## Abstract 2

We present a novel enzyme-triggered fluorogenic prodrug, termed AcKLP, that incorporates dual-locked enzyme activation, ensuring that the prodrug remains inactive until it confronts the unique enzymatic environment of glioblastoma cells.

## Graphical abstract

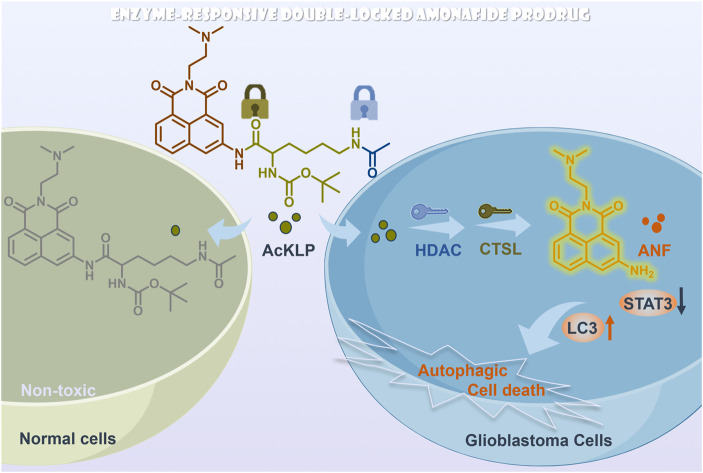

## 1. Introduction

Glioblastoma (GBM) is an aggressive tumor that originates in the brain or spinal cord.^1,2^ Its characteristic genetic profile favors a high level of mitotic activity and angiogenesis while retaining the ability to migrate from precursor cells and astrocytes, which can infiltrate into the cortical tissue and lead to metastasis in other regions of the central nervous system.^3–5^ As such, GBM represents one of the hardest cancers to cure, with a median overall survival of only 15 months after diagnosis. Surgical resection with the assistance of chemotherapy is the main method used for GBM therapy.^6–9^ The alkylating agent temozolomide (TMZ) is the standard chemotherapeutic agent used against GBM due to its established efficacy.^10^ However, the acquisition of resistance to TMZ is common among patients, which limits its clinical use.^11^ In this context, amonafide (ANF), a naphthalene dicarboximide, has long been recognized as a small-molecule anticancer drug. ANF is a topoisomerase II (Topo II) inhibitor and DNA intercalator that induces apoptosis by inhibiting Topo II binding to double-stranded DNA and retains anticancer activity even in the presence of multi-drug resistance and has been investigated in phase II clinical trials for the treatment of gliomas.^12–14^ Unfortunately, it was found that ANF has some major pharmacological limitations, including notable side effects.^15^ These adverse properties have driven the focus toward the design and synthesis of prodrugs to improve therapeutic outcomes.

Enzymes over-expressed in cancer cells provide important opportunities for designing prodrugs with enzyme-cleavable linkages.^16,17^ These prodrugs can be activated in response to specific enzyme activities, allowing on-demand release of drugs and improved cancer selectivity.^18^ In recent years, responsive prodrugs have been extensively developed that can be activated selectively in the presence of bioactive small molecules or single enzymes upregulated in diseased tissues.^19–21^ However, since cellular functions are regulated by multiple active molecules as well as the microenvironment, dual stimuli-responsive systems can offer enhanced cancer therapy specificity. Histone deacetylases (HDACs) are conserved enzymes that regulate various cellular processes by removing acetyl groups from lysine residues on histones and non-histone proteins.^22^ Alterations in HDAC expression play an important role in the pathogenesis of cancers and have been linked to tumor progression.^23^ Similarly, cathepsin L (CTSL), a lysosomal protease, is involved in a wide range of cellular functions and has an important impact on disease development.^24^ The upregulation of CTSL is a marker of cancer progression and metastasis, holding both prognostic and diagnostic value.^25^ Notably, both HDACs and CTSL are abnormally upregulated in GBM. Yet to date, no HDAC- and CTSL-responsive dual-locked amonafide prodrug for glioblastoma treatment has been developed. Specifically, our approach could potentially enhance the specificity and efficacy of GBM therapy by targeting these upregulated enzymes.

**Scheme 1:**
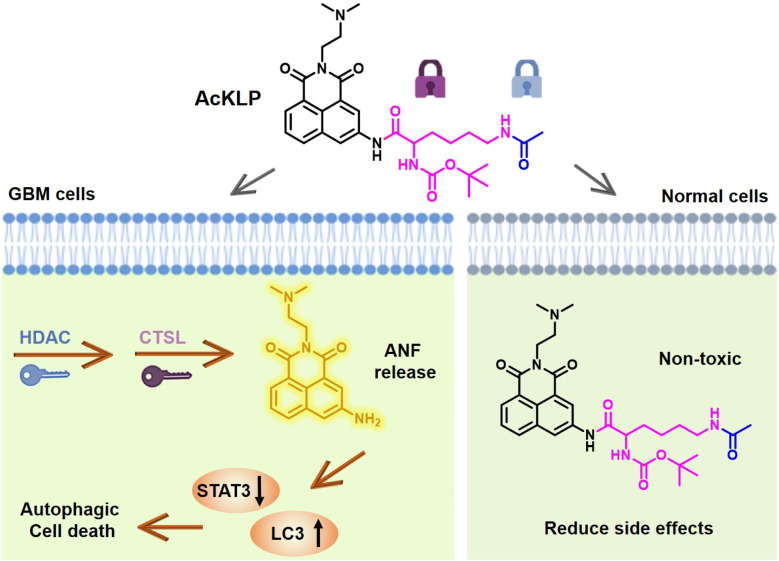
Schematic illustration of the mechanism of enzyme-responsive double-locked amonafide prodrug AcKLP for the treatment of glioblastoma (GBM) with minimal side effects.

In this research, an enzyme-responsive double-locked prodrug, designated AcKLP, was designed and synthesized based on the aforementioned considerations. AcKLP is comprised of the anticancer drug amonafide (ANF) and short peptides that are specifically recognized by HDAC and CTSL. Following HDAC-mediated deacetylation of the short peptide, the lysine fragment is exposed and subsequently selectively cleaved by CTSL to release the drug ANF. Importantly, *in vitro* results confirmed that AcKLP significantly reduced the adverse effects of ANF on normal cells. For instance, the IC_50_ in human umbilical vein endothelial cells (HUVEC) was higher than 100 μM, compared to an IC_50_ of approximately 0.8 μM for ANF-treated HUVEC. Importantly, the fluorescent prodrug AcKLP exhibited potent cytotoxicity in human malignant glioblastoma cells (IC_50_ for U87, ∼2.3 μM). Detailed molecular mechanism evaluation suggested that AcKLP primarily induces autophagic death of U87 cells. The antiproliferative activity of AcKLP was further confirmed by 3D multicellular U87 tumor spheroid assays and *in vivo* anti-tumor studies, highlighting its potential to reduce toxicity while retaining good antitumor activity. Therefore, the fluorogenic prodrug AcKLP developed in this research may provide a strategy for the treatment of glioblastoma with minimal side effects (Scheme 1).

## 2. Results and discussion

### 2.1. Design and synthesis

**Fig. 1:**
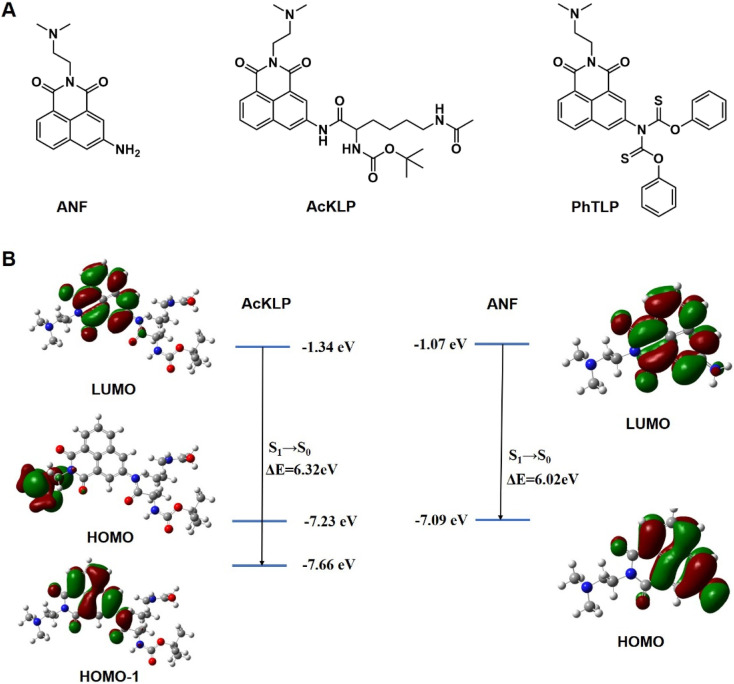
(A) Chemical structures of amonafide (ANF), HDAC- and CTSL-responsive double-locked amonafide prodrug AcKLP, and cysteine-responsive control prodrug PhTLP. (B) Electron cloud distribution of AcKLP and ANF. The ground-state geometries were fully optimized using density functional theory (DFT) at the CAM-B3LYP/6-31G(d,p) level of theory.

Inspired by the combination of HDAC and CTSL activities for sequential unmasking in drug activation reported by Ueki *et al.*,^26^ we synthesized an HDAC- and CTSL-responsive double-locked amonafide fluorescent prodrug AcKLP (Fig. 1). The synthetic route towards AcKLP was straightforward (Scheme S1†), and the detailed characterization studies are included in the ESI.† The absorption and fluorescence spectra of AcKLP are given in Fig. S1.† The stability of AcKLP in media was assessed in PBS by UV-vis spectroscopy, according to a previously described protocol. After 48 hours of incubation, the peak shape and position remained unchanged, suggesting that AcKLP is structurally stable in the media (Fig. S2†). Additionally, we synthesized a cysteine-responsive amonafide prodrug, PhTLP, as a control compound (Scheme S1†), which was designed based on the specific nucleophilic addition reaction between thiobenzoate and cysteine.^27^ The cysteine-triggered drug release was studied by spectroscopic techniques, including absorption and emission spectroscopy (Fig. S3 and S4†). The findings confirmed the potential of AcKLP as a stable, responsive prodrug system, and the cysteine-responsive PhTLP confirmed the importance of the dual locked AcKLP system.

Furthermore, the theoretical basis for fluorescence change was verified using calculations of the electron cloud density and the energy level differences of LUMO and HOMO between AcKLP and ANF. As depicted in Fig. 1B, the emission peak of AcKLP with the highest oscillator strength originates from the LUMO → HOMO−1 transition, exhibiting a calculated energy gap (Δ*E*) of 6.32 eV. Conversely, the emission peak of ANF with the greatest oscillator strength is attributed to the LUMO → HOMO transition, featuring a calculated energy gap (Δ*E*) of 6.02 eV. The reduced energy gap indicates that the emission of ANF generated after the enzymatic reaction exhibits a red shift compared to the prodrug AcKLP, which is consistent with the fluorescence emission spectrum of AcKLP and ANF (Fig. S1†).

### 2.2. *In vitro* cytotoxicity

To evaluate the cytotoxicity of these two fluorescent prodrugs, we evaluated their antiproliferative activity against a panel of human cancer cell lines (HCT-116, A549, MCF-7, HeLa, and U87), and one normal cell line (HUVEC) using the CCK-8 assays, with the anti-cancer drug ANF as the control. The results, presented as IC_50_ values, are given in Table 1, based on three parallel experiments. The IC_50_ value of AcKLP against U87 cells was determined to be 2.26 μM, demonstrating similar antitumor activity to ANF (IC_50_, 3.10 μM). For other cancer types, the cell viability of AcKLP was slightly lower than that of ANF-treated cells, which might be attributed to the incomplete cleavage of the prodrug in these cells. Notably, AcKLP exhibited a significantly reduced cytotoxic effect towards normal HUVEC (IC_50_ > 100 μM) compared to ANF (IC_50_, 0.80 μM), with the cancer selectivity index (CSI) values for AcKLP and ANF being >44.25 and 0.25, respectively. In contrast, the IC_50_ values of the prodrug PhTLP for both cancer and normal cells were similar, indicating that the cysteine-responsive prodrug PhTLP lacked selective cytotoxicity towards cancer cells. Taken together, these findings suggest that AcKLP can selectively target enzyme-positive cells and is therefore suitable for the treatment of GBM. Based on these results, our fluorescent prodrug AcKLP was subjected to additional biological assays.

**Table 1:** Cytotoxicity (IC_50_, μM) of the investigated compounds in various cell lines^a^

Cell line	ANF	AcKLP	PhTLP
U87	3.10 ± 0.2	2.26 ± 0.1	19.24 ± 1.2
HCT-116	4.50 ± 0.3	15.85 ± 1.2	17.63 ± 1.1
A549	5.70 ± 0.3	24.30 ± 1.6	35.08 ± 2.0
MCF-7	2.40 ± 0.1	4.70 ± 0.4	10.20 ± 0.8
HeLa	8.80 ± 0.5	19.10 ± 1.5	13.20 ± 0.6
HUVEC	0.80 ± 0.04	>100	9.04 ± 0.5
CSI	0.25	>44.25	0.47

a CSI (cancer selectivity index) value = IC_50_ (HUVEC)/IC_50_ (U87). Human cancer cells (HCT116, A549, MCF-7, HeLa, and U87) and human umbilical vein endothelial cells (HUVEC) were treated with ANF, AcKLP, and PhTLP for 48 h. Data are expressed as mean ± SD from three independent experiments.

### 2.3. *In vitro* drug release studies

**Fig. 2:**
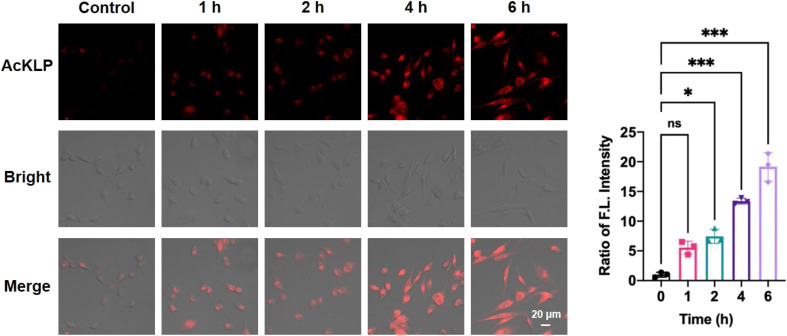
Confocal microscopy images of U87 cells treated with 5 μM AcKLP for different times (*λ*_ex_ = 405 nm, *λ*_em_ = 550–650 nm). All the data represent the average of three independent experiments with an error bar of ±standard deviation (SD). The fluorescence intensity of control was set as 1. Statistical analyses were performed with a two-tailed Student's *t*-test with unequal variance, *p-value < 0.05, ***p-value < 0.001.

To investigate the activation of the prodrug AcKLP, we used fluorescence imaging in live U87 cells. As shown in Fig. 2, red fluorescence was observed in live cells and increased over 6 hours, confirming the release of ANF. Moreover, the behavior of drug release was validated and confirmed using LC-MS assays (Fig. S5†). Co-localized fluorescence imaging revealed that the fluorescence signal generated by the prodrug co-localized with lysosomes (Fig. S6†), suggesting that the prodrug primarily targets lysosomes and releases ANF after being activated therein, which may be due to CTSL, a lysosomal protease, being involved in the activation process. Besides, when U87 cells were incubated with AcKLP in the presence of a histone deacetylase inhibitor (SAHA) or a cathepsin L inhibitor (E64D), the fluorescence signal intensities were attenuated (Fig. S7†). These findings indicate that the release of ANF from AcKLP occurs in cancer cells as a result of the combined actions of histone deacetylases and cathepsin L. To evaluate its selective release in tumor cells, we further incubated the prodrug AcKLP in normal cells (HUVEC), and the results indicated that normal cells treated with AcKLP exhibited only weak fluorescence (Fig. S8†). In addition, the cellular uptake of AcKLP was analyzed by HPLC. As shown in Fig. S9,† lysates of U87 cells treated with AcKLP for 6 h confirmed that AcKLP could be effectively converted into ANF. In marked contrast, no ANF product was detected in the lysates of HUVEC cells. These observations confirmed the lysosomal targeting and activation process of AcKLP, providing insight into its potential effectiveness as a prodrug for cancer treatment.

### 2.4. Anticancer mechanism of fluorescent prodrug AcKLP

**Fig. 3:**
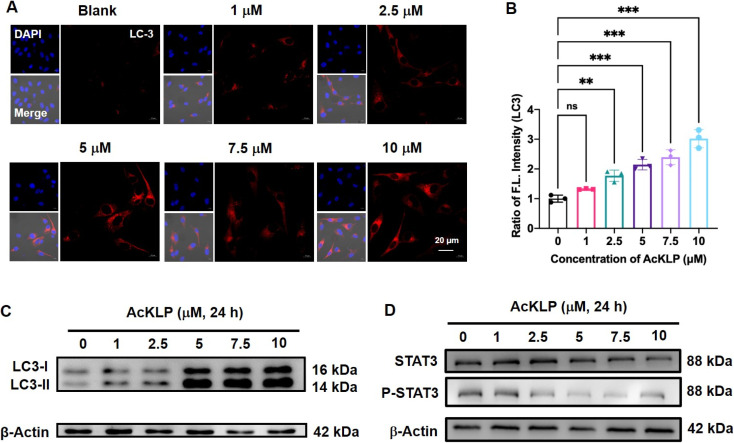
(A) Fluorescence imaging of U87 cells treated with different concentrations of AcKLP, stained for the LC-3 antibody. The LC-3 channel is shown in red, *λ*_ex_ = 561 nm, *λ*_em_ = 570–680 nm. (B) Quantification of relative fluorescence intensities in (A). The fluorescence intensity of cells treated with 0 μM AcKLP was set as 1. Data are presented as mean ± S.D., *n* = 3. **p-value < 0.01, ***p-value < 0.001. (C) Expression levels of LC3-I and LC3-II and (D) expression levels of STAT3 and p-STAT3 in U87 cells treated with the indicated doses of AcKLP for 24 hours, as determined by western blotting. β-Actin was used as a protein loading control.

Autophagy, a lysosome-dependent cellular degradation process responds to a variety of environmental and cellular stresses. Given that the prodrug AcKLP primarily targets lysosomes and releases ANF, we thus investigated whether AcKLP induces autophagy. The microtubule-associated protein 1A/1B-light chain 3 (LC3)-phosphatidylethanolamine conjugate (LC3-II), formed by the conjugation of the cytosolic form of LC3 (LC3-I) to phosphatidylethanolamine, is recruited to autophagosomal membranes and serves as a specific biomarker of autophagy. We first examined the expression of LC3 by immunofluorescence. As shown in Fig. 3A and B, red fluorescence was observed, and it increased in a concentration-dependent manner, indicating that the prodrug AcKLP induced a high expression level of LC3 and the formation of autophagosomes. The ratio of LC3-II/LC3-I has been verified as a specific marker of autophagy. To further confirm the ability of AcKLP to induce autophagy, we performed a Western blot analysis. As illustrated in Fig. 3C and S10,† a concentration-dependent increase in the conversion of LC3-I to LC3-II was observed in AcKLP-treated cells. In addition, the STAT3 signaling pathway is implicated in many aspects of the autophagic process. The protein expression levels were thus detected to determine whether AcKLP could inhibit the STAT3 pathway. As shown in Fig. 3D and S11,† the STAT3 expression and phosphorylation were inhibited after treatment with AcKLP in U87 cells for 24 h. To further validate the mechanism of AcKLP, the expression of the classical receptor of autophagy, the p62 protein, was examined. The results show that the content of p62 was downregulated in the AcKLP group, while the ANF group exhibited no significant change compared to the blank group (Fig. S12†). Certainly, AcKLP has apoptosis-inducing effect to some degree owning to the partial transferring of the activated ANF from the lysosome to the nucleus (Fig. S13†). To better understand the location of activated ANF, we incubated ANF or AcKLP with U87 cells for different durations and performed co-localization confocal imaging with nuclear dyes (Hoechst 33342). Analysis of cell images indicated that in the ANF group, most ANF fluorescence was observed in the nucleus, and the AcKLP group showed some degree of colocalization of red and blue fluorescence at 12 h and 24 h, suggesting that the activated ANF is transferred from the lysosome to the nucleus (Fig. S14†). Taken together, these results suggest that AcKLP induces autophagic cell death in U87 cells, highlighting its potential as an effective anticancer agent through the modulation of autophagy and STAT3 signaling pathways, which differs from its precursor ANF.

### 2.5. Cytotoxicity in 3D multicellular tumor spheroids

**Fig. 4:**
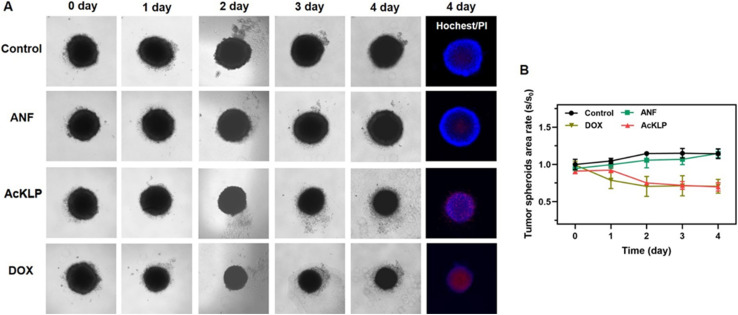
(A) Representative pictures of 3D U87 tumor spheroids treated with ANF (20 μM), AcKLP (20 μM) and DOX (2 μM) at different times. The blue color (Hoechst 33342, *λ*_ex_ = 405 nm, *λ*_em_ = 420–500 nm) marks the healthy cells, and the red color (propidium iodide, *λ*_ex_ = 561 nm, *λ*_em_ = 600–640 nm) indicates the dead cells. (B) The volume changes of 3D tumor spheroids under different conditions with an extended incubation time from 0 to 4 days. Error bars: S.D., *n* = 3.

To further validate the anti-tumor effect of AcKLP, we used three-dimensional (3D) multicellular tumor spheroids (MCTSs). These spheroids, which typically exhibit oxygen gradients at diameters ranging from 300 to 500 μm and central hypoxia, serve as a model to simulate solid tumors.^28^ The performance of the AcKLP treatment was then evaluated. As shown in Fig. 4A and B, after 4 days of incubation with 20 μM AcKLP or 2 μM doxorubicin (DOX), the spheroids began to disintegrate and lose their original structure, indicating that AcKLP effectively disrupted the integrity of the tumor spheroids. Furthermore, staining of the spheroids with propidium iodide (PI) revealed that the dead cells in the AcKLP-treated group emitted red fluorescence (Fig. 4A). These results indicated that AcKLP inhibits the growth of U87 cells in a three-dimensional tumor model, demonstrating its potential efficacy in more complex tumor microenvironmental systems.

### 2.6. *In vivo* antitumor effects of AcKLP

**Fig. 5:**
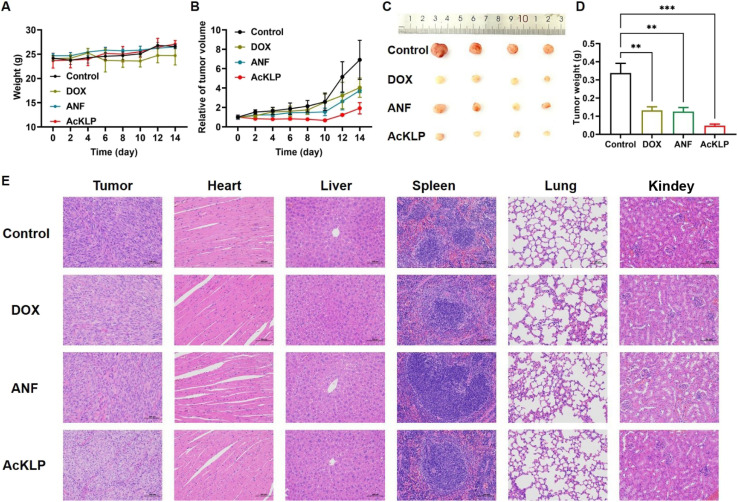
(A) Body weight changes in each group. (B) Relative tumor volumes of different treated groups after the indicated treatment. (C) Photograph of dissected tumors after the treatment. (D) Tumor weights in each group. (E) Representative photos of hematoxylin and eosin (H&E) stained sections of each group after treatment (200× magnification).

Given the potential anti-tumor effect of AcKLP at the cellular level, we further evaluated the *in vivo* therapeutic efficacy of AcKLP in a subcutaneous xenograft U87 tumor-bearing mouse model. All mice were randomly divided into four groups (*n* = 5 in each group) and injected intravenously with DOX, ANF, AcKLP, or PBS, respectively. The body weights and relative tumor volumes of the U87 tumor-bearing mice were recorded to assess the therapeutic effect *in vivo*. As shown in Fig. 5A, the body weight of the mice in all groups exhibited a trend of general growth within 14 days, suggesting good biocompatibility and low side effects of AcKLP. Tumor growth curves exhibited rapid and uncontrollable tumor growth in the control group (Fig. 5B), while moderate tumor inhibition was observed in mice treated with DOX and ANF. Despite DOX demonstrating significant efficacy against U87MG cells *in vitro*, factors such as tumor angiogenesis and the presence of glioblastoma stem cells may influence its effectiveness *in vivo*.^29^ In contrast, the AcKLP group exhibited significant tumor growth inhibition, which might be attributed to the dual enzyme-responsive nature of AcKLP. After 14 days of treatment, the tumor tissues were collected and weighed (Fig. 5C and D). After comparing the results of tumor photographs and weight, a relative inhibition rate of approximately 72.4% was observed, indicating that AcKLP inhibited tumor growth more significantly than observed in the DOX and ANF groups. Additionally, hematoxylin and eosin (H&E) staining images revealed obvious features of cell damage, such as reduced nuclear volume and cell number (Fig. 5E), further demonstrating the excellent anti-tumor effect of the AcKLP group. Moreover, no significant pathological changes were observed in the H&E images of major organs after treatment, suggesting good biosafety of AcKLP in both *in vitro* and *in vivo* models.

## 3. Conclusions

In summary, we have developed a strategy to mitigate the adverse effects of amonafide (ANF) by using an enzyme-responsive double-locked fluorescent prodrug. As a proof of concept, we synthesized AcKLP, an HDAC- and CTSL-responsive double-locked amonafide prodrug, and our *in vitro* results indicated that it significantly attenuated the adverse effects of ANF on normal cells. Further mechanistic studies confirmed that AcKLP induced autophagic cell death in U87 cells. Importantly, AcKLP was effective in disrupting three-dimensional multicellular tumor spheroids and also exhibited potential *in vivo* tumor growth inhibition in a xenograft U87 tumor-bearing mouse model, highlighting the anti-glioma potential. We anticipate that this enzyme-responsive double-locked amonafide prodrug may provide a new therapeutic avenue for the treatment of glioblastoma.

## 4. Experimental section

### 4.1. Materials and methods

Tin(ii) chloride (SnCl_2_), hydrochloric acid (HCl), 3-nitro-1,8-naphthalic anhydride, 1,1-dimethylethylenediamine, phenyl chlorothionocarbonate, *N*-Boc-*N*-acyl lysine, *N*-(3-dimethyl aminopropyl)-*N*′-ethylcarbodiimide hydrochloride (EDCI), 1-(3-dimethylaminopropyl)-3-ethylcarbodiimide hydrochloride (DMAP) and Boc-Lys (AC)-OH were purchased from Bidepharm (Shanghai, China). All anhydrous and absolute solvents were obtained from J&K Scientific Ltd. Additional chemical reagents were purchased from Beijing Innochem Science & Technology Co., Ltd.

Column chromatography was performed using silica gel 60 (200–300 mesh) from Qingdao Haiyang Chemical Co. Ltd. ^1^H and ^13^C NMR spectra were recorded on a 400 MHz Bruker Avance NMR spectrometer (Germany) using CDCl_3_, DMSO-*d*_6_, and methanol-*d*_4_ as solvents. Electrospray ionization mass spectroscopy (ESI-MS) data were acquired using an LCQ mass spectrometer (Thermo Scientific). UV-vis absorption spectra and fluorescence emission spectra were measured using a Lambda 365 UV-vis spectrophotometer and an FS5 spectrofluorometer, respectively.

### 4.2. Synthesis of fluorescent prodrugs

#### 4.2.1. Synthesis of amonafide (ANF)

ANF was synthesized following previously reported methods.^15^ Briefly, 3-amino-1,8-naphthalic anhydride (300 mg, 1.40 mmol) and 1,1-dimethylethylenediamine (186 mg, 2.11 mmol) were dissolved in 6.5 mL of ethanol. The suspension was heated under reflux for approximately 2.5 h. The mixture was then allowed to cool slowly to room temperature. The resulting precipitate was collected by vacuum filtration and washed with 3 mL of ethanol. The obtained yellow solid was dried to a constant weight in a vacuum drying oven (280 mg, yield, 71.0%): ^1^H NMR (400 MHz, methanol-*d*_4_) *δ* 8.17 (dd, *J* = 7.2, 0.8 Hz, 1H), 8.00 (d, *J* = 2.3 Hz, 1H), 7.95 (d, *J* = 8.1 Hz, 1H), 7.62–7.54 (m, 1H), 7.33 (d, *J* = 2.3 Hz, 1H), 4.35–4.27 (m, 2H), 2.77–2.70 (m, 2H), 2.42 (s, 6H).

#### 4.2.2. Synthesis of *tert*-butyl(6-acetamido-1-((2-(2-(dimethylamino)ethyl)-1,3-dioxo-2,3-dihydro-1*H*-benzo[*de*]isoquinolin-5-yl)amino)-1-oxohexan-2-yl)carbamate (AcKLP)

Boc-Lys (AC)-OH (100 mg, 0.353 mmol), EDCI (203.1 mg, 1.059 mmol), and DMAP (86.0 mg, 0.703 mmol) were added to a reaction flask, which was then purged with nitrogen. Anhydrous dichloromethane was added until the solid dissolved. The solution was stirred in an ice-water bath for 10 min, after which a solution of ANF (100 mg) in dichloromethane was added dropwise. The resulting mixture was stirred at room temperature for 48 hours and monitored by TLC. Upon completion of the reaction, the mixture was poured into H_2_O (150 mL) and extracted with dichloromethane (3 × 50 mL). The organic phase was sequentially washed with saturated NaHCO_3_ (2 × 30 mL) and saturated NaCl, dried over anhydrous sodium sulfate, and concentrated under reduced pressure to afford the crude product. The crude compound was purified by silica gel column chromatography to obtain a faint yellow compound, AcKLP (43 mg, yield, 39.0%). ^1^H NMR (400 MHz, CDCl_3_) *δ* 9.43 (s, 1H), 8.85 (d, *J* = 9.3 Hz, 1H), 8.51–8.38 (m, 2H), 8.11 (t, *J* = 8.6 Hz, 1H), 7.76–7.64 (m, 1H), 5.82 (d, *J* = 5.8 Hz, 1H), 5.43 (t, *J* = 6.2 Hz, 1H), 4.33 (t, *J* = 7.0 Hz, 3H), 3.43–3.23 (m, 2H), 2.68 (dd, *J* = 14.8, 7.8 Hz, 2H), 2.39 (s, 6H), 2.04 (s, 3H), 1.90–1.74 (m, 4H), 1.63 (dd, *J* = 13.5, 6.7 Hz, 2H), 1.51 (s, 9H). ^13^C NMR (101 MHz, methanol-*d*_4_) *δ* 172.74, 171.79, 163.66, 163.18, 156.89, 137.07, 133.29, 131.77, 129.00, 126.96, 124.05, 123.51, 122.11, 121.46, 121.28, 79.46, 56.16, 55.64, 44.31 (3C), 38.85, 37.15, 31.71, 28.78, 27.52, 23.18, 21.27. ESI-MS: C_29_H_39_N_5_O_6_ [M + H]^+^, calculated: 554.2900, found: 554.2990.

#### 4.2.3. Synthesis of *O*-phenyl(2-(2-(dimethylamino)ethyl)-1,3-dioxo-2,3-dihydro-1*H*-benzo[*de*]isoquinolin-5-yl)(phenoxy carbonothioyl)carbamothioate (PhTLP)

ANF (300 mg, 1.06 mmol) was dissolved in 10 mL of tetrahydrofuran (THF), followed by the addition of phenyl chlorothionocarbonate (270 μL, 1.56 mmol). The mixture was stirred at room temperature under a nitrogen atmosphere for 24 hours. After this period, the reaction mixture was concentrated under reduced pressure. The resulting residue was purified by column chromatography to obtain a light-yellow solid (80 mg, yield, 43.0%). ^1^H NMR (400 MHz, CDCl_3_) *δ* 8.76 (d, *J* = 2.1 Hz, 1H), 8.66 (d, *J* = 7.3 Hz, 1H), 8.39 (d, *J* = 2.1 Hz, 1H), 8.28 (d, *J* = 7.6 Hz, 1H), 7.87–7.81 (m, 1H), 7.48 (t, *J* = 7.9 Hz, 4H), 7.35 (t, *J* = 7.4 Hz, 2H), 7.26–7.22 (m, 4H), 4.42 (t, *J* = 6.9 Hz, 2H), 2.83 (t, *J* = 6.7 Hz, 2H), 2.49 (s, 6H). ^13^C NMR (101 MHz, CDCl_3_) *δ* 190.22, 163.89, 163.44, 153.72, 142.32, 134.15, 132.86, 132.34, 132.21, 131.54, 129.82, 127.89 (2C), 127.68, 126.99, 124.49, 122.83, 121.71 (2C), 56.53, 45.23, 31.95, 31.52, 30.14, 29.72, 29.39, 22.72, 14.16. ESI-MS: C_30_H_25_N_3_O_4_S_2_ [M + H]^+^, calculated: 556.1286, found: 556.1382.

### 4.3. Fluorescence analysis

Solutions of AcKLP (10 μM) and ANF (10 μM) in PBS solution (10 mM, pH 7.4, 1% DMSO) were analyzed using a UV spectrophotometer and a fluorescence spectrometer. The response of the probe PhTLP (10 μM) to Cys in PBS buffer (10 mM, pH 7.4, 1% DMSO, and 1 mM CTAB) was measured by both fluorescence and UV spectrophotometry, including UV absorption, excitation, and emission spectra.

### 4.4. Stability study

The stability of AcKLP was assessed using UV-vis spectroscopy. A solution of AcKLP (10 μM) in PBS solution (10 mM, pH 7.4, 1% DMSO) was prepared, and its absorbance was recorded at different time intervals over a 48 hour period at 25 °C.

### 4.5. Cell culture

Cell lines (HCT116, A549, MCF-7, HeLa, HUVEC, and U87) were cultured in Dulbecco's Modified Eagle's Medium (DMEM) (Gibco Company, USA), supplemented with 1% penicillin–streptomycin and 10% fetal bovine serum (FBS). The cultures were maintained in a 5% CO_2_ atmosphere at 37 °C.

### 4.6. Cell viability assay

The cytotoxicity of the fluorescent prodrugs AcKLP and PhTLP was evaluated using the Cell Counting Kit-8 (CCK-8) assay in both normal and tumor cells. Cells were seeded in 96-well plates at a density of 5000 cells per well and incubated in a water-saturated incubator with 5% CO_2_ at 37 °C for 12 hours. Subsequently, the prodrugs AcKLP and PhTLP were added at varying concentrations (0.01, 0.1, 1, 10, and 100 μM). After an additional 48 h of incubation, 100 μL of CCK-8 reagent was added to each well, and the plates were incubated for another 4 h at 37 °C. Afterward, the cell viability was determined by measuring the absorbance at 450 nm using a microplate reader. Each concentration was tested in triplicate wells.

### 4.7. Cellular confocal imaging

To verify the responsiveness of prodrug AcKLP, U87 cells were subjected to fluorescence signal analysis after different treatments. The U87 cells were seeded into 12-well plates at a density of 2 × 10^4^ cells per well and incubated for 12 hours. Following this, the cells were incubated with AcKLP for 0, 1, 2, 4, and 6 h. After incubation, the cells were washed three times with PBS and imaged using a Carl Zeiss LSM 880 confocal fluorescence microscope. Fluorescence signals from different treatments were quantitatively analyzed using ImageJ software.

### 4.8. Immunofluorescence assays

U87 cells in the logarithmic growth phase were plated into 12-well plates (2 × 10^4^ cells per well) and incubated for 12 h. The cells were then treated with various concentrations of the prodrug AcKLP (1 μM, 2.5 μM, 5 μM, 7.5 μM, and 10 μM) for another 24 h. After treatment, the cells were washed three times with PBS buffer and fixed with 3.7% formaldehyde for 15 min at room temperature. Afterward, the cells were washed again with PBS, permeabilized with 0.2% Triton X-100 for 15 min, and incubated with the anti-LC3 rabbit polyclonal antibody overnight. Then, the working buffer was discarded, and the cells were washed twice with PBS and incubated with FITC-labeled goat-anti-rabbit lgG for 1 h in the dark. After washing three times with PBS, the cells were stained with DAPI for 5 min. Finally, the samples were analyzed using confocal fluorescence microscopy. DAPI staining (blue) was observed with an excitation wavelength of 405 nm and an emission range of 430–490 nm, while the LC3 channel (red) was observed with an excitation wavelength of 561 nm and an emission range of 570–680 nm.

### 4.9. Western blot analysis

U87 cells were treated with various concentrations of the probe AcKLP (1, 2.5, 5, 7.5, and 10 μM) and then lysed in RIPA buffer (Beyotime, Shanghai, China) on ice for 30 minutes. Then, the mixture was centrifuged at 13 000*g* for 15 min at 4 °C, and the supernatant was collected. Protein concentrations were determined using the BCA protein assay kit (EpiZyme, Shanghai, China). Proteins were separated by SDS-PAGE (Bio-Rad) according to the molecular weight of the target proteins and transferred onto PVDF membranes (Millipore, Bedford, MA, USA). Membranes were blocked with 5% non-fat milk at room temperature for 1 hour and then incubated with primary antibodies overnight at 4 °C. After washing four times with 1× TBST, membranes were incubated with peroxidase-conjugated secondary antibodies (anti-rabbit IgG or anti-mouse IgG) at room temperature for 1 hour. Signals were detected using an enhanced chemiluminescence reagent (Thermo Scientific, Rockford, IL, USA) and visualized with a Tanon-4600SF imaging system. The protein levels were normalized to β-actin and quantified using ImageJ software. Antibodies were sourced as follows: LC-3 and Stat3 from Proteintech, Inc., USA; Phospho-Stat3 (Try705) from Cell Signaling Technology Inc., USA.

### 4.10. 3D multicellular tumor spheroid culture

U87 cells were seeded into a U-shaped 96-well plate at a density of 6 × 10^4^ cells per well and cultured at 37 °C with 5% CO_2_ for 5 days to form a 3D multicellular tumor spheroid with a radius of 500 μm. The cell culture medium was replaced with fresh medium every two days.

### 4.11. 3D multicellular tumor spheroid imaging

Five-day-old multicellular tumor spheroids (MCTSs) were treated with ANF (20 μM), AcKLP (20 μM), and DOX (2 μM). The spheroid growth was monitored using an inverted fluorescence microscope (Brightfield, Olympus, RVL2-K, Echo). After four days, the MCTSs were stained with 5 μL of propidium iodide (PI) and 5 μL of Hoechst 33342 for 30 min at 37 °C and then imaged using an inverted fluorescence microscope (Hoechst 33342, *λ*_ex_ = 405 nm, *λ*_em_ = 420–500 nm; propidium iodide, *λ*_ex_ = 561 nm, *λ*_em_ = 600–640 nm).

### 4.12. *In vivo* treatment

For the tumor-bearing BALB/c nude mouse model, a total of twenty male BALB/c nude mice, aged 5 weeks and weighing between 18 and 20 g, were procured directly from Changzhou Cavens Laboratory Animal Co., Ltd, in complete compliance with the Changzhou University's Guide for the Care and Use of Laboratory Animals and has been reviewed by the Laboratory Animal Ethics Committee. The U87 tumor-bearing nude mouse model was established through the subcutaneous injection of U87 cells (1 × 10^7^) into the right flank of each BALB/c nude mouse. When the tumor reached a volume of 100 mm^3^, the mice were randomly divided into four groups (*n* = 4) and subsequently administered with intravenous injections of DOX (1 mg kg^−1^), ANF (1 mg kg^−1^), AcKLP (1 mg kg^−1^), and PBS (as a control) every two days. Tumor volumes and body weights were monitored throughout the study. Tumor volumes and body weights were measured every two days for 14 days. The volume was calculated based on the following equation: *LB*^2^/2 (*L* means length and *B* means width). On the 15th day of the treatment, the tumors and major organs were sectioned for further analysis.

## Data availability

The data that support the findings of this study are available in the ESI† of this article.

## Author contributions

Y. Qian conceived the idea and designed the research; W. C., X. J. L., and R. M. X. performed the chemical research; B. Z. performed the biological research; W. C. and Y. L. Y. performed the *in vitro* spectroscopy tests of the probe; C. W. S., W. W. S., and X. B. Z. contributed new reagents/analytical tools; W. C., X. J. L., and B. Z. analyzed the data and wrote the original draft; Y. Q. supervised the study. All authors contributed to the manuscript's original editing. W. C., X. J. L., B. Z., Y. Q., and T. D. J. wrote and revised the manuscript.

## Conflicts of interest

The authors declare no conflict of interest.

## Supplementary Material

SC-OLF-D4SC04555F-s001
