# Supplementary material for: An enzyme-responsive double-locked amonafide prodrug for the treatment of glioblastoma with minimal side effects
Source: Chem Sci. 2024 Nov 1;15(46):19336–44. doi: 10.1039/d4sc04555f (PMC11528907; doi:10.1039/d4sc04555f)
Supplement: SC-015-D4SC04555F-s001 [file SC-015-D4SC04555F-s001.pdf]

Supplementary Information (SI) for Chemical Science.
This journal is © The Royal Society of Chemistry 2024

Supporting Information

Enzyme-responsive double-locked amonafide prodrug for the treatment of glioblastoma with low side effects

Wei Cheng[†, a], Yanli Yang[†, a], Bo Zhang[†, *, b], Chen-Wen Shao[†, d], Wei Chen[†, a], Ruimin Xia[a], Wenwei Sun[a], Xiubo Zhao[b], Bing Zhang [c], Xiangjie Luo[*, a], Tony D. James[*, e, f], Yong Qian[*, a, c, e]

[b]*Jiangsu Collaborative Innovation Center of Biomedical Functional Materials, School of Chemistry and Materials Science, Nanjing Normal University, Nanjing 210046, China*

[b]*School of Pharmacy, Changzhou University, Changzhou 213164, China*

[c]*Department of Radiology, Nanjing Drum Tower Hospital, The Affiliated Drum Tower Hospital of Nanjing University Medical School, Nanjing 210008, China*

[d]*Wuxi School of Medicine, Jiangnan University, Wuxi 214122, China*

[e]*Department of Chemistry, University of Bath, Bath BA2 7AY United Kingdom*

[f]*School of Chemistry and Chemical Engineering. Henan Normal University, Xinxiang 453007 China*

[†]*These authors contributed equally to this work.*

Table of contents

1. Supplemental Figures 3

2. NMR and MS Spectra 11

1. Supplemental Figures

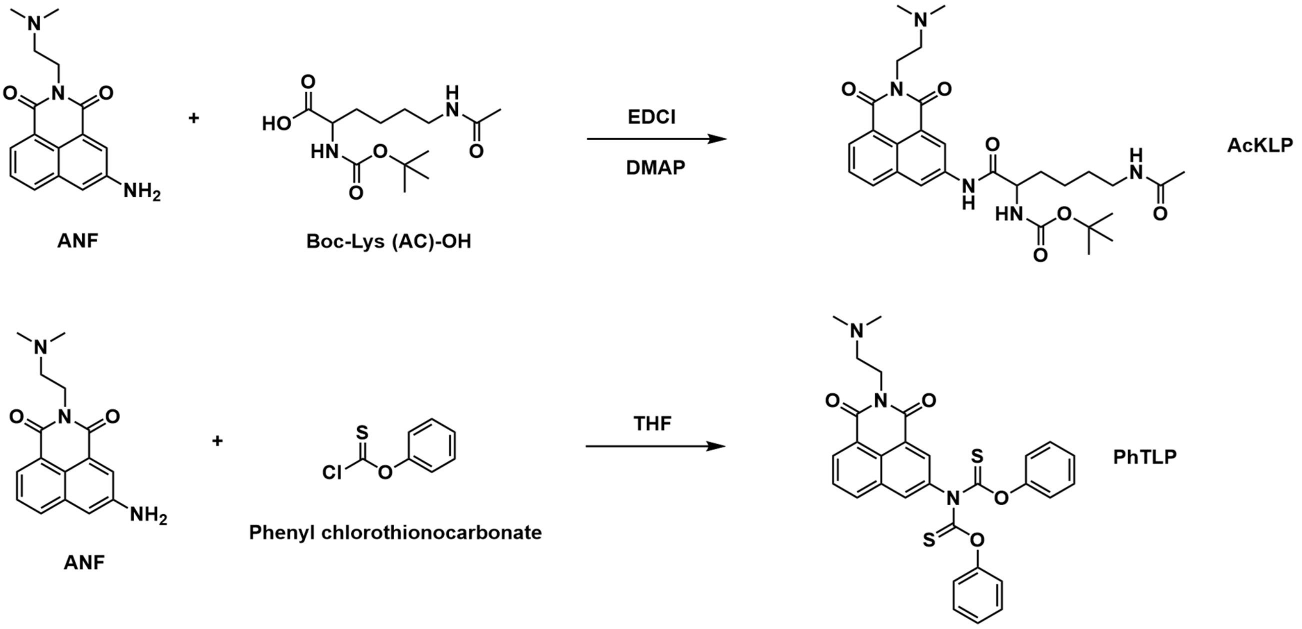

Scheme S1. Synthesis routes of **AcKLP** and **PhTLP**.

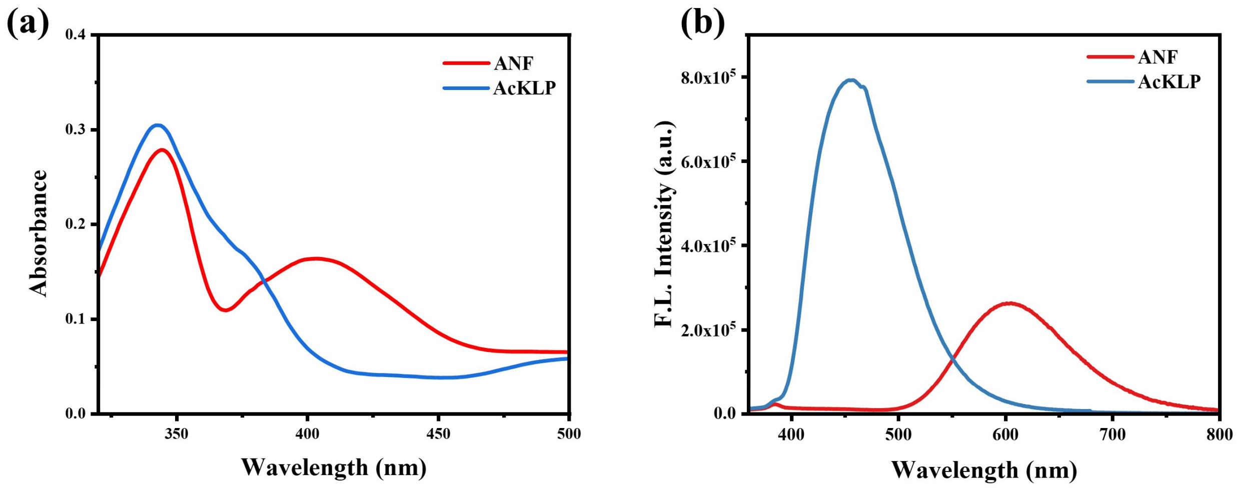

Fig. S1. Absorption (a) and fluorescence emission (b) spectra of **AcKLP** and ANF (10 μM) in a PBS (10 mM, pH 7.4, 1%DMSO) buffer (AcKLP, $\lambda_{ex}/\lambda_{em}$ =340 nm/485 nm).

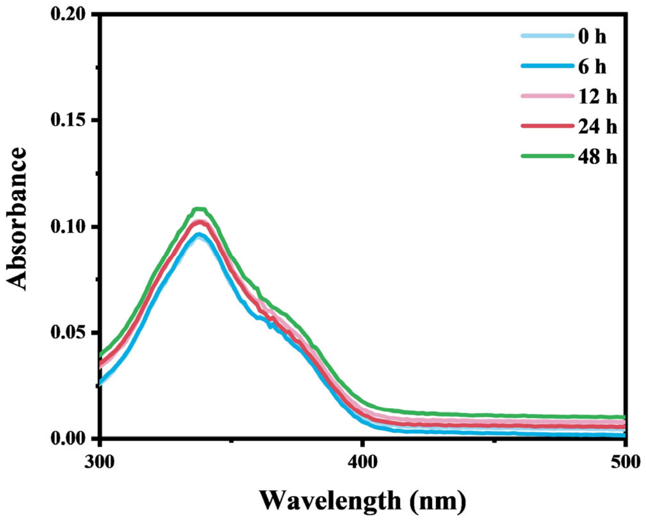

Fig. S2. UV-vis spectra of **AcKLP** (10 μM) in a PBS (10 mM, pH 7.4, 1% DMSO) buffer recorded for a period of 48 h at 298 K.

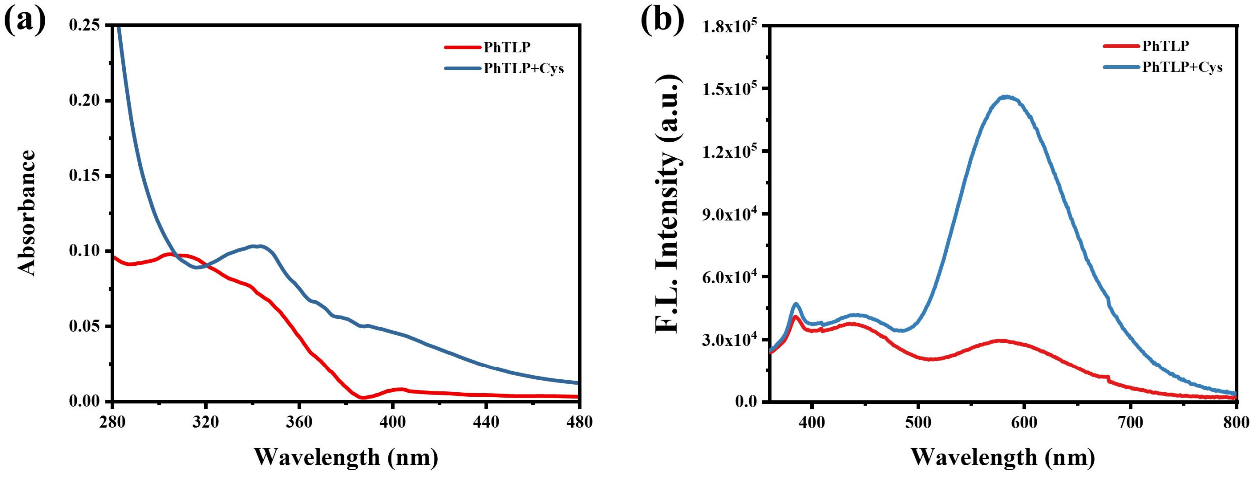

Fig. S3. Absorption (a) and fluorescence emission (b) spectra of **PhTLP** (10 μM) before and after addition of Cys (100 μM) to PBS (10 mM, pH 7.4, 1%DMSO) and 1 mM CTAB buffer (**PhTLP**, $\lambda_{ex}/\lambda_{em}$ =340 nm/585 nm).

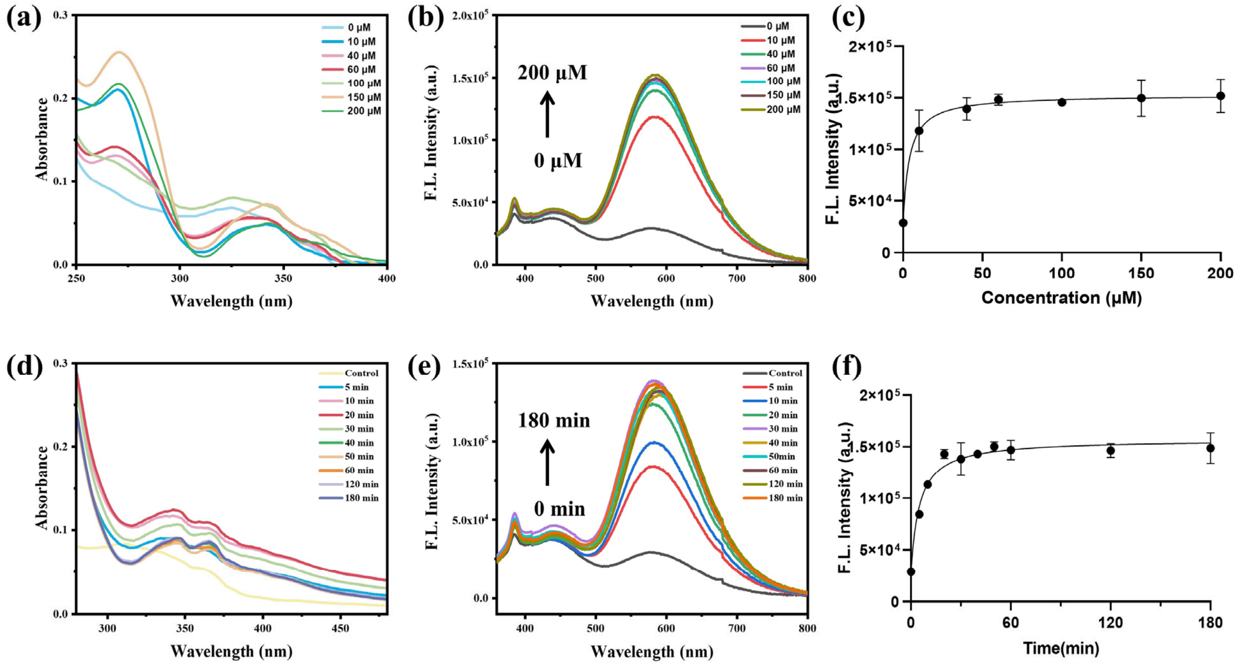

Fig. S4. The change of **PhTLP** (10 μM) absorption (a) and fluorescence intensity (b) with time (0-180 min) in the presence of Cys (100 μM). (c) The time-dependent fluorescence intensity of **PhTLP** (10 μM) at 585 nm in the presence of Cys (100 μM). Absorption (d) and fluorescence intensity (e) of **PhTLP** (10 μM) incubated with different concentrations of Cys (1-200 μM).

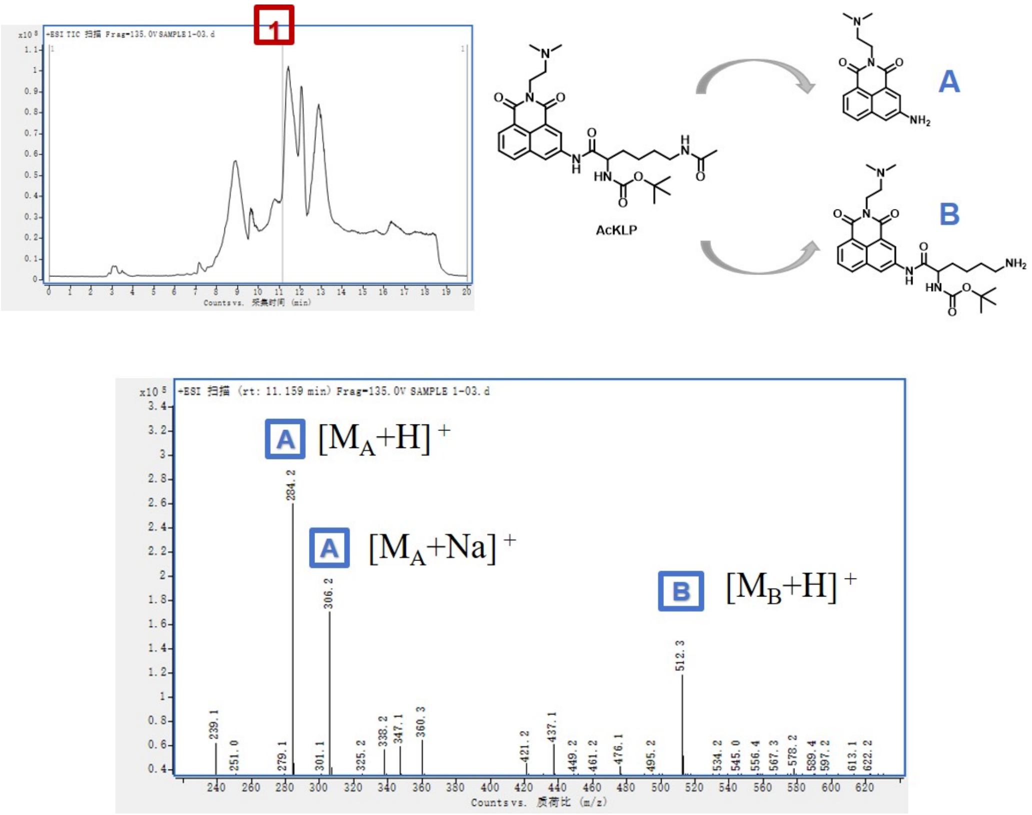

Fig. S5. The release behavior of intracellular **AcKLP** was analyzed by LC-MS. U87 cells were treated with **AcKLP** (5 μM) for 48 h. Cells were then lysed with cell lysis solution and the supernatant was extracted. Followed by the addition of 0.5 mL of methanol to the supernatant to precipitate the protein, the supernatant was taken for LC-MS analysis.

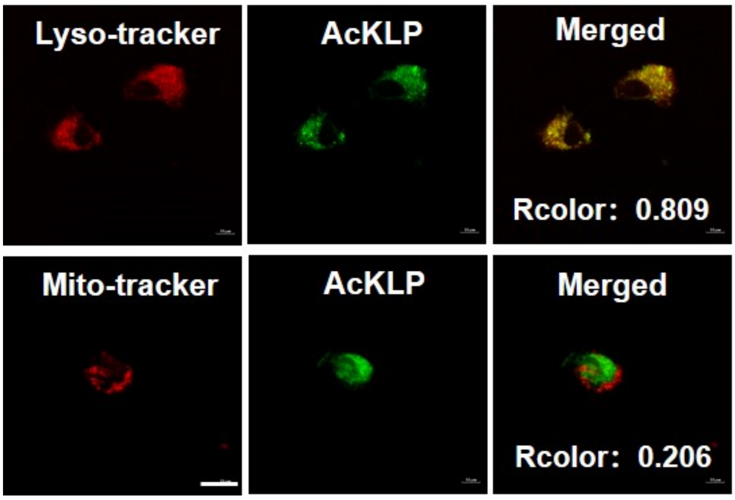

Fig. S6. Confocal microscopy images of U87 cells treated with **AcKLP** for 6 h, respectively. **AcKLP** (5 μM, λ_{ex} = 405 nm, λ_{em} range 550−650 nm); MitoTracker Red (0.3 μM, λ_{ex} = 561 nm, λ_{em} range 570−620 nm); LysoTracker Green (2 μM, λ_{ex} = 488 nm, λ_{em} range 500−550 nm). Scar bar: 20 μm.

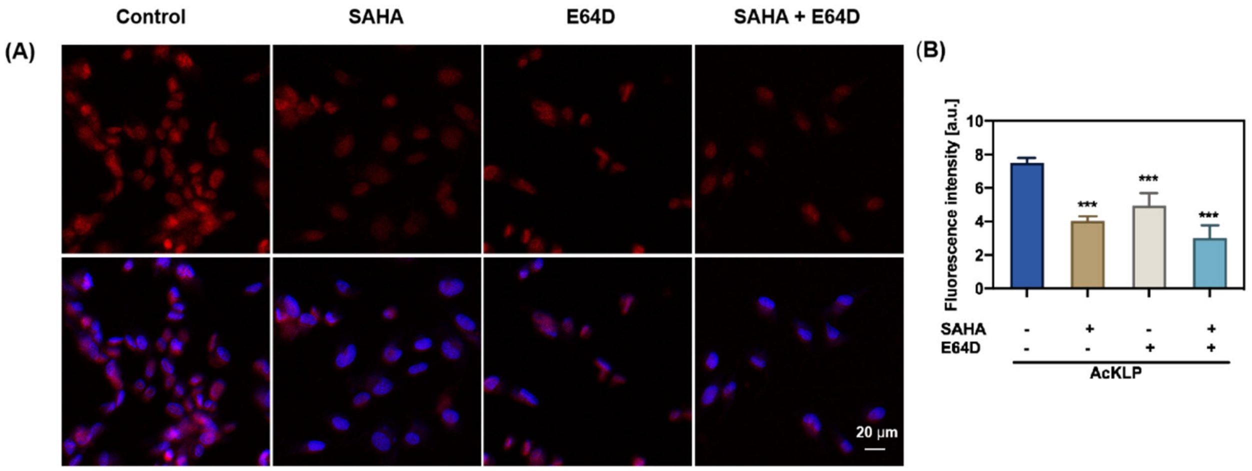

Fig. S7. (A) U87 cells were pre-incubated with 50 μM histone deacetylases inhibitor SAHA (2 h) or 50 μM cathepsin L inhibitor E64D (0.5 h), followed by treatment with 5 μM **AcKLP** for 6 h. Cell images were obtained using confocal fluorescence microscopy (scale bar = 20 μm). (B) Quantification of relative fluorescence intensities in (A). Data are presented as mean ± S.D., n = 3. ***p -value < 0.001.

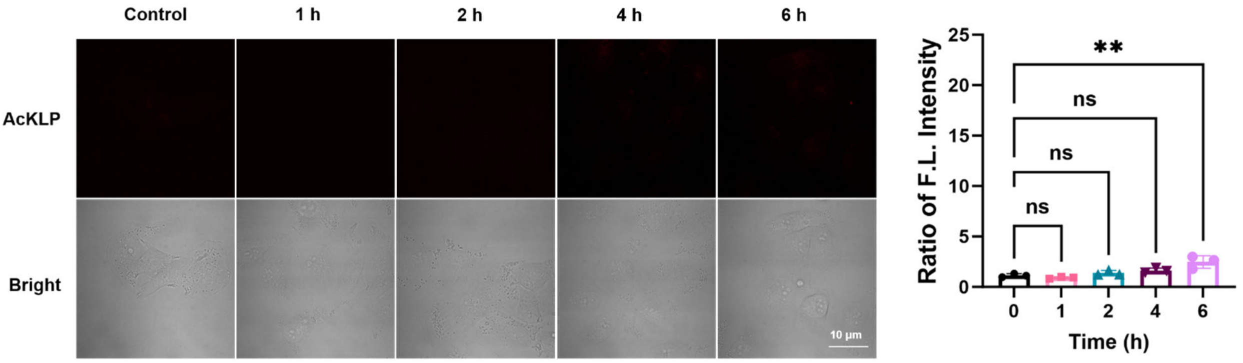

Fig. S8. Confocal microscopic images of HUVEC cells treated with 5 μM AcKLP for different times (λ_{ex} = 405 nm, λ_{em} = 550−650 nm). All the data represent the average of three independent experiments with an error bar of ± standard deviation (SD).

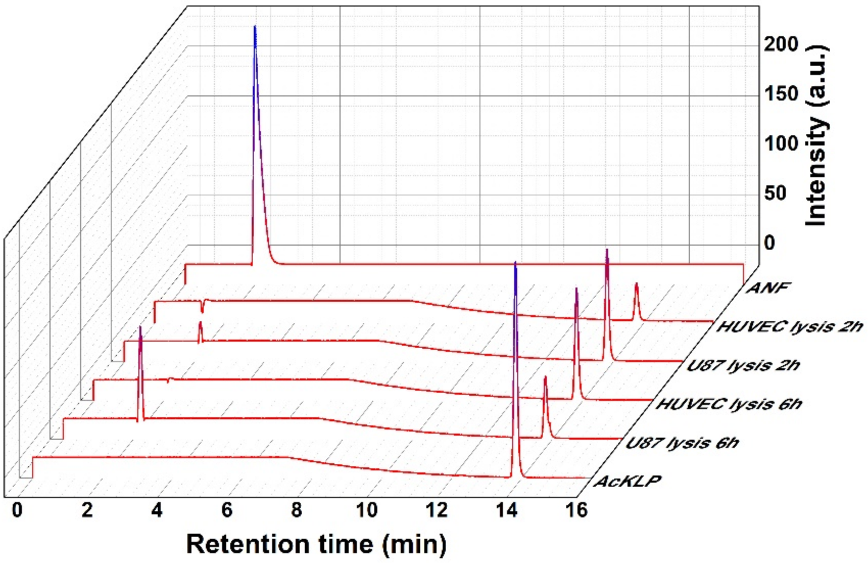

Fig. S9. U87 or HUVEC cells, incubated with 10 μM **AcKLP** for 2 and 6 h, were lysed, and the supernatant was removed for analysis by HPLC (detection at 340 nm).

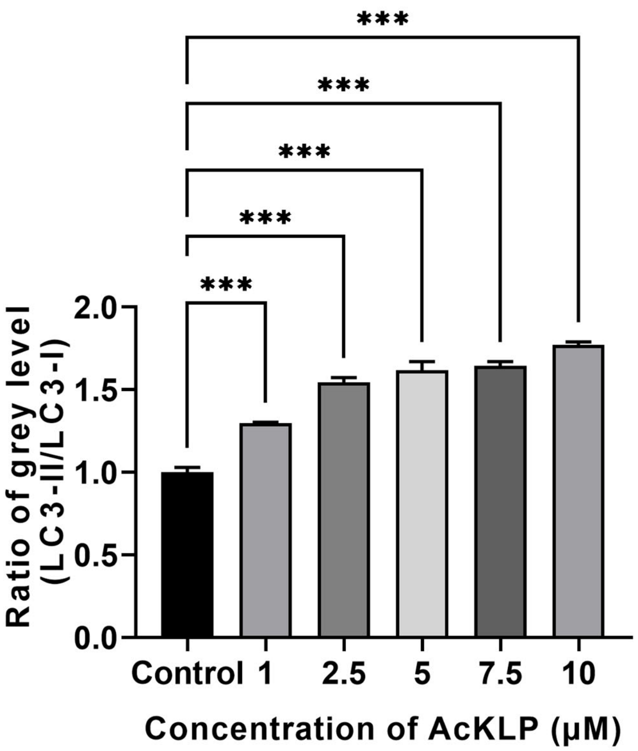

Fig. S10. Relative grey intensity of LC-3 is based on the result of Fig 3C. Error bars are ± S.D. (n = 3).

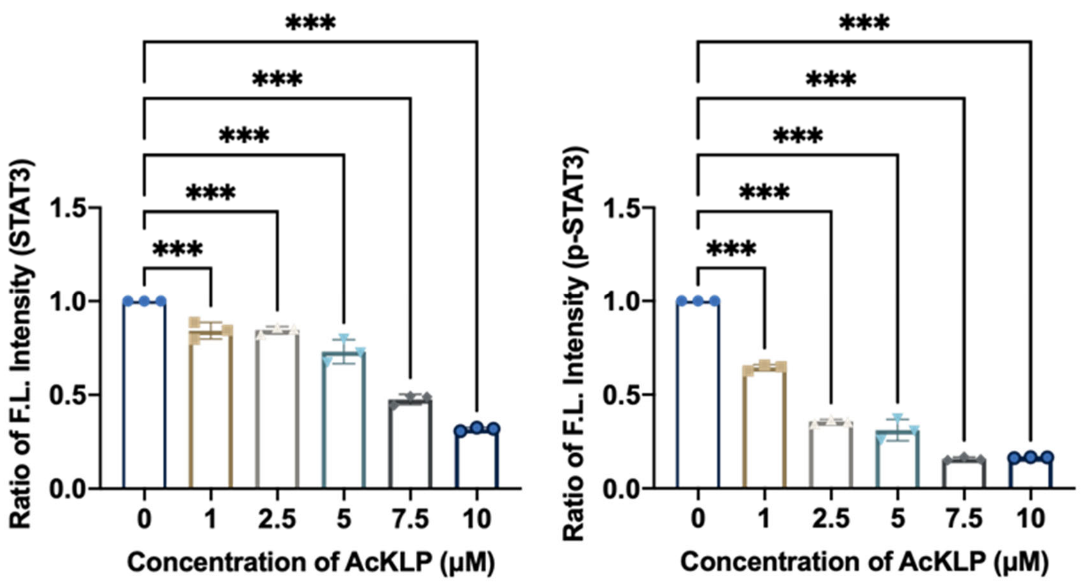

Fig. S11. The relative ratio of the grey levels of STAT3 and p-STAT3 is based on the result of Fig 3D. The grey level of 0 μM AcKLP-treated cells was set as 1, respectively. Statistical analyses were performed using two-way ANOVA with multiple comparisons, $^{***}P < 0.001$ *vs*. 0 μM **AcKLP** group. Error bars are ± S.D. (n = 3).

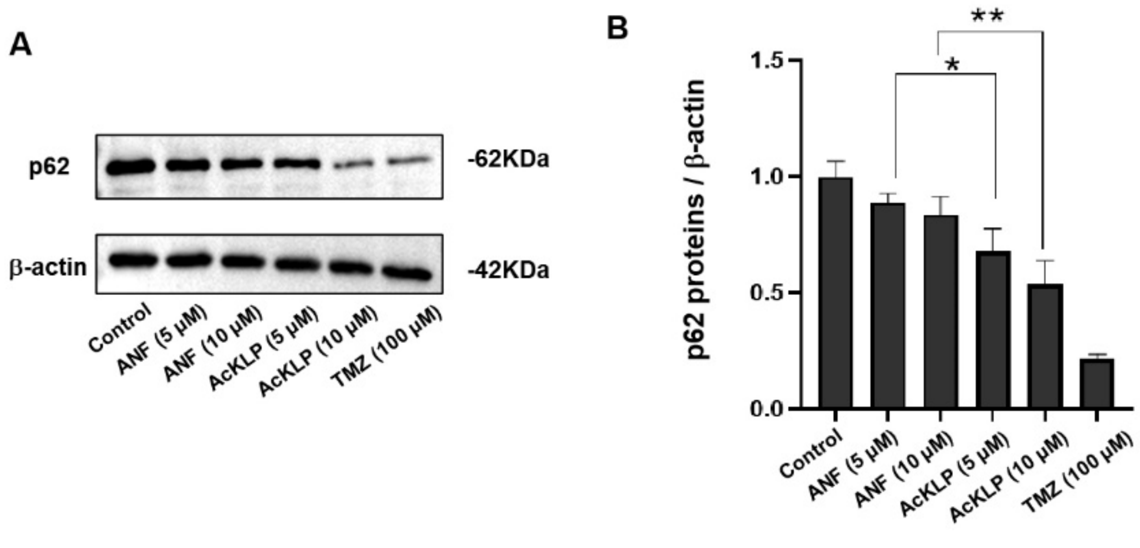

Fig. S12. (A) Effects of ANF and **AcKLP** on the protein expression of p62 in U87 cells after 24 h treatment. (B) The relative grey intensity of p62 is based on the result of (A). The fluorescence intensity of cells treated with 0 μM **AcKLP** was set as 1. Data are presented as mean ± S.D., n = 3. $^{**}p$-value < 0.01, $^{***}p$ -value < 0.001. Error bars are ± S.D. (n = 3).

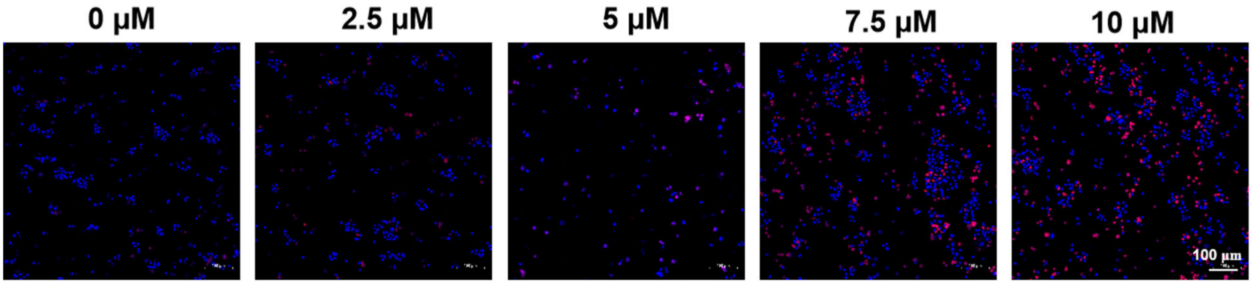

Fig. S13. U87 cells stained by Hochest 33342 and PI after being treated with different concentrations of **AcKLP** for 24 h. The blue color (Hochest 33342, λ_{ex} = 405 nm, λ_{em} = 420−500 nm) indicates healthy cells, and the red color (propidium iodide, λ_{ex} = 561 nm, λ_{em} = 600−640 nm) indicates dead cells. Scale bar, 100 μm.

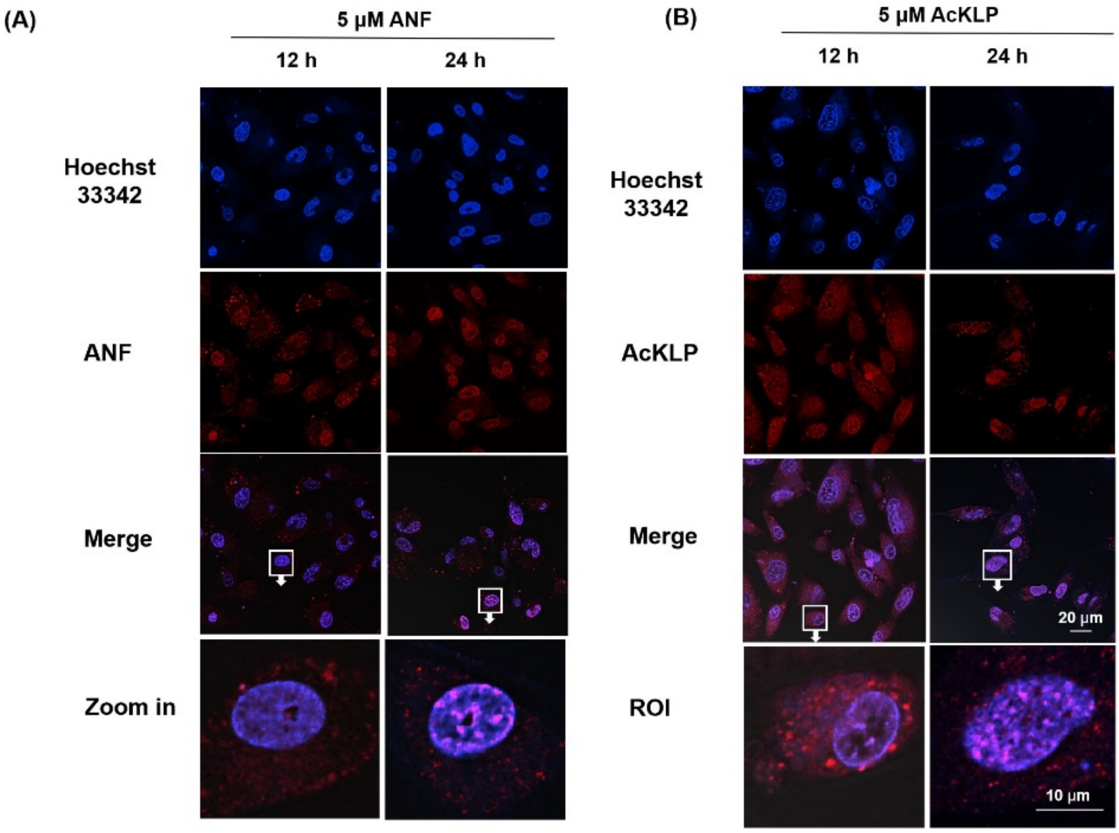

Fig. S14. U87 cells were treated with ANF and **AcKLP** (5 μM) for 12 and 24 h. The cells were imaged and analyzed after incubation with Hoechst 33342 and Lyso-Tracker Red DND-99. ANF, $\lambda_{ex}/\lambda_{em}$ = 405/550-650nm; Hoechst 33342, $\lambda_{ex}/\lambda_{em}$ = 405/425−475 nm. Lyso-Tracker Red DND-99, $\lambda_{ex}/\lambda_{em}$ =577/590 nm. Scale bar = 25 μm.

2. NMR and MS Spectra

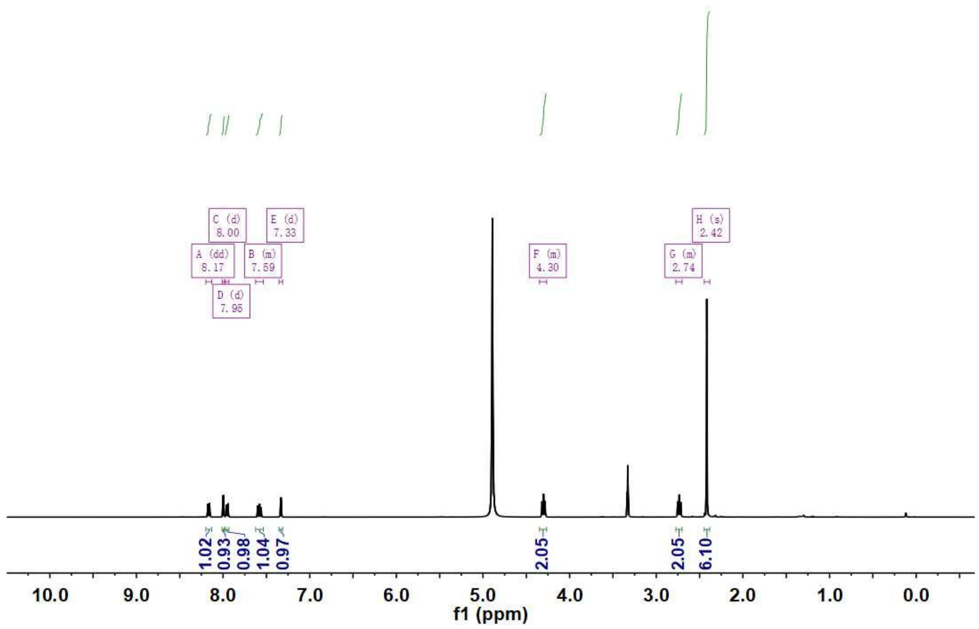

Fig. S15. ^{1}H NMR of compound **ANF**.

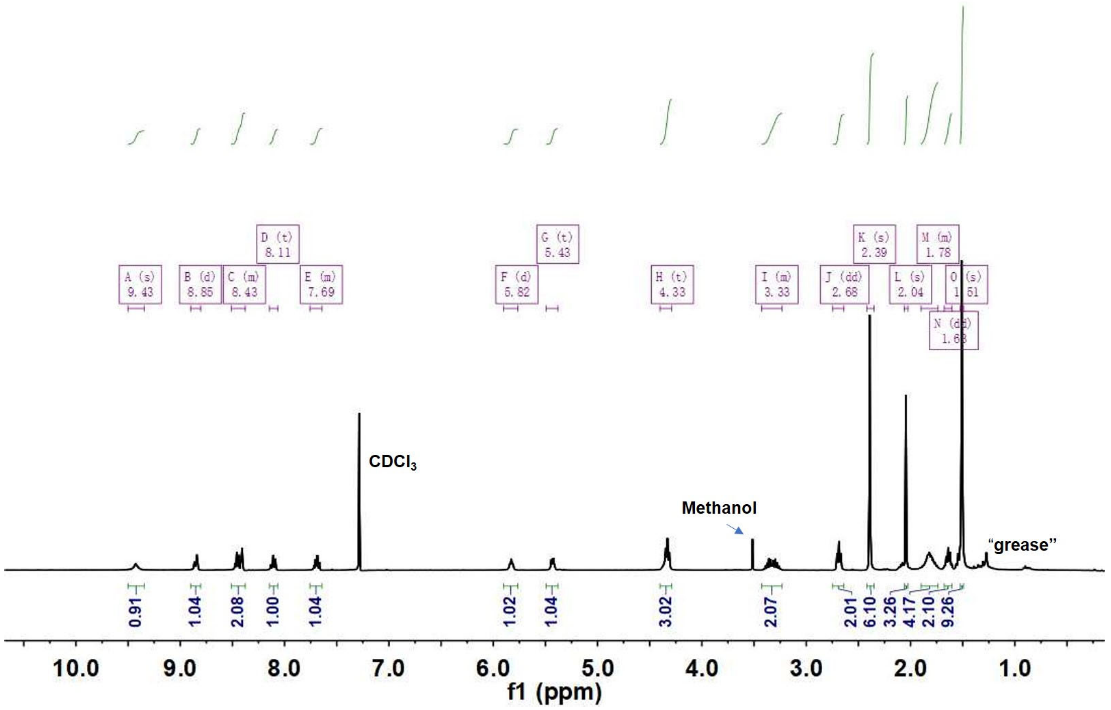

Fig. S16. ^{1}H NMR of compound **AcKLP**.

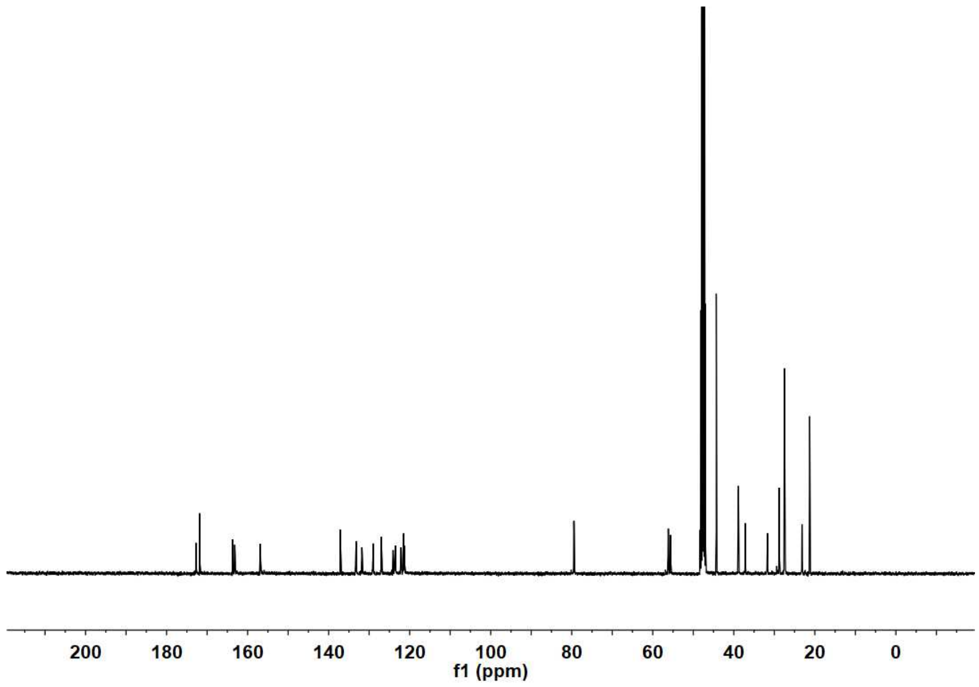

Fig. S17. ^{13}C NMR of compound **AcKLP**.

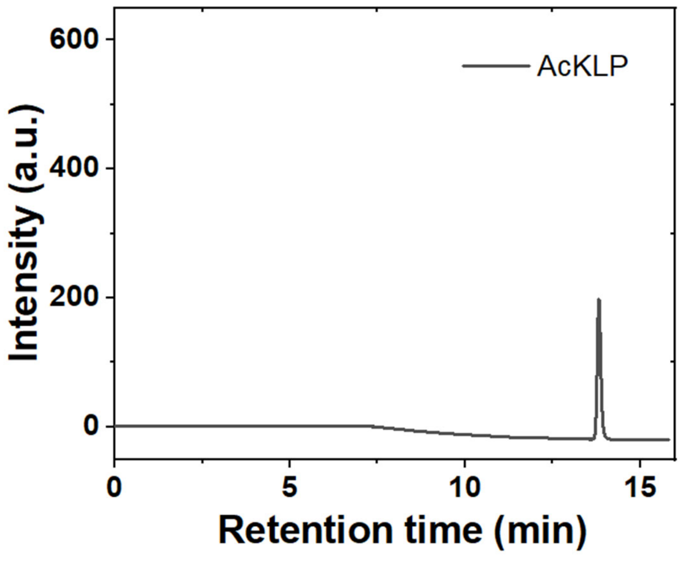

Fig. S18. HPLC analyzes the purity of **AcKLP**.

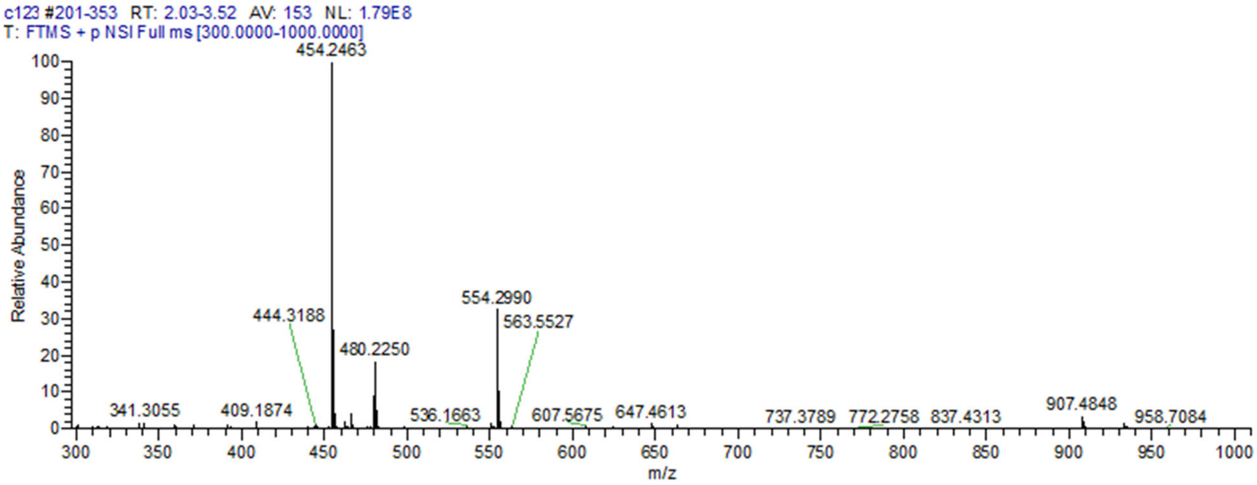

Fig. S19. MS spectrum of compound **AcKLP**.

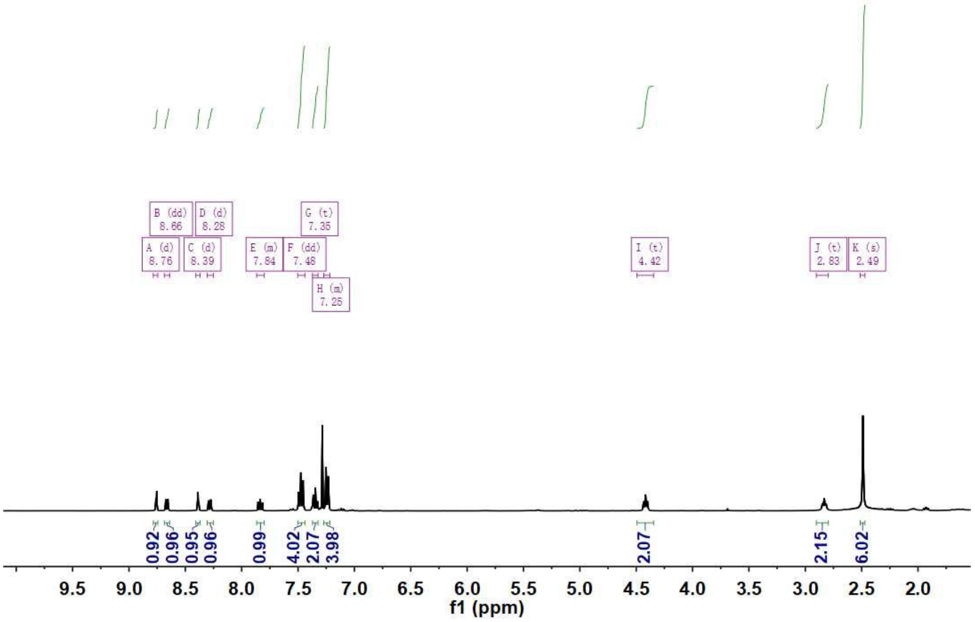

Fig. S20. ^{1}H NMR of compound **PhTLP**.

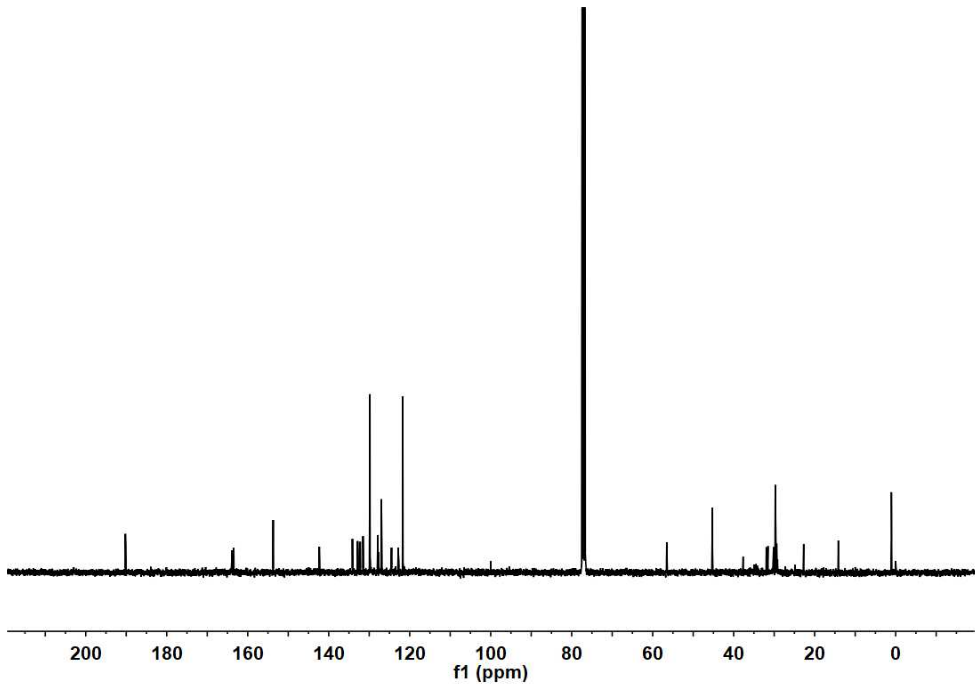

Fig. S21. ^{13}C NMR of compound **PhTLP**.

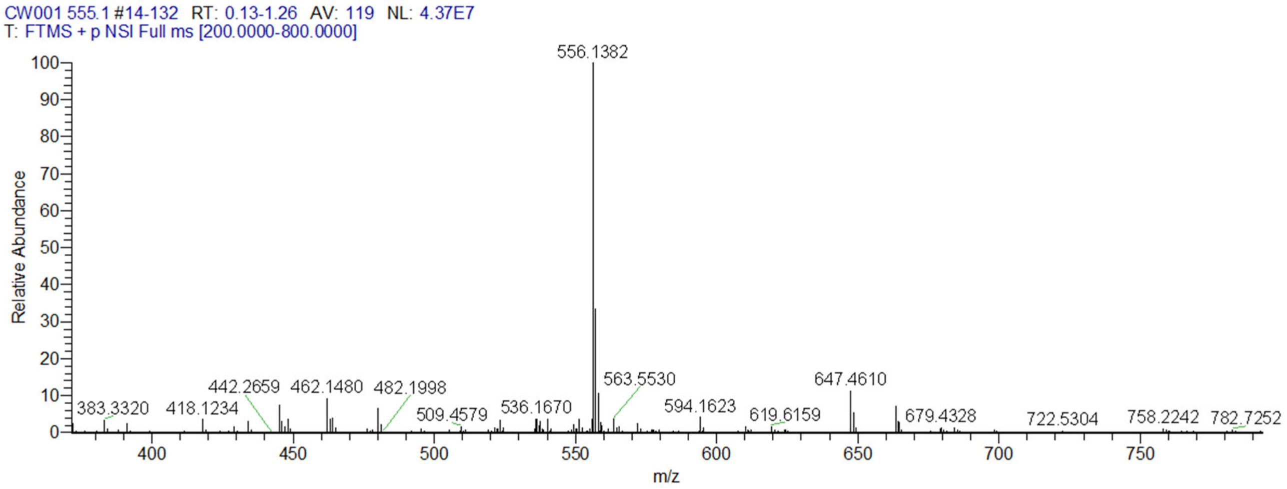

Fig. S22. HRMS spectrum of **PhTLP**.
